# Data Mining of Molecular Simulations Suggest Key Amino Acid Residues for Aggregation, Signaling and Drug Action

**DOI:** 10.3390/biom11101541

**Published:** 2021-10-19

**Authors:** Vaibhav Gurunathan, John Hamre, Dmitri K. Klimov, Mohsin Saleet Jafri

**Affiliations:** 1 BASIS Independent Silicon Valley, San Jose, CA 95126, USA; vaibhav.gurunathan@gmail.com; 2 School of Systems Biology, George Mason University, Fairfax, VA 22030, USA; jhamre@gmu.edu (J.H.III); dklimov@gmu.edu (D.K.K.); 3 Center for Biomedical Engineering and Technology, University of Maryland School of Medicine, Baltimore, MD 21201, USA

**Keywords:** amyloid beta, Alzheimer’s disease, molecular simulation, machine learning, cerebral amyloid angiopathy

## Abstract

Alzheimer’s disease, the most common form of dementia, currently has no cure. There are only temporary treatments that reduce symptoms and the progression of the disease. Alzheimer’s disease is characterized by the prevalence of plaques of aggregated amyloid β (Aβ) peptide. Recent treatments to prevent plaque formation have provided little to relieve disease symptoms. Although there have been numerous molecular simulation studies on the mechanisms of Aβ aggregation, the signaling role has been less studied. In this study, a total of over 38,000 simulated structures, generated from molecular dynamics (MD) simulations, exploring different conformations of the Aβ42 mutants and wild-type peptides were used to examine the relationship between Aβ torsion angles and disease measures. Unique methods characterized the data set and pinpointed residues that were associated in aggregation and others associated with signaling. Machine learning techniques were applied to characterize the molecular simulation data and classify how much each residue influenced the predicted variant of Alzheimer’s Disease. Orange3 data mining software provided the ability to use these techniques to generate tables and rank the data. The test and score module coupled with the confusion matrix module analyzed data with calculations of specificity and sensitivity. These methods evaluating frequency and rank allowed us to analyze and predict important residues associated with different phenotypic measures. This research has the potential to help understand which specific residues of Aβ should be targeted for drug development.

## 1. Introduction

Alzheimer’s disease is a progressive neurodegenerative disease in which patients experience symptoms such as impairment of behavioral and cognitive functions such as memory, comprehension, language, attention, reasoning, and judgment [1]. Alzheimer’s is the most common form of dementia [1]. There are three types of Alzheimer’s disease: early onset, late onset, and familial Alzheimer’s disease (FAD) [2]. While FAD only accounts for about 25% of Alzheimer’s cases, it can provide valuable insight in the alterations in Aβ function [3]. Alzheimer’s is currently the sixth leading cause of death in the United States. Furthermore, there was a sharp increase in the number of Alzheimer’s cases over the last two decades, emphasizing the need for an effective treatment for Alzheimer’s disease. The current treatments available only address the symptoms of Alzheimer’s but not the underlying cause.

Small aggregates of Aβ called oligomers are cytotoxic and are thought to play a role in neurodegeneration in Alzheimer’s disease [4]. The oligomers can form protofibrils. Aβ protofibrils aggregate together to form plaques which disrupt the neuronal connections in the brain. Many studies have explored drug interventions to prevent Aβ aggregation [4,5]. However, clinical trials of anti-aggregations drugs have thus far mostly failed suggesting that researchers might consider targeting other mechanisms to treat Alzheimer’s disease [5]. Five drugs that target Aβ to treat Alzheimer’s disease are currently in clinical trials (or have recently completed clinical trials): gantenerumab, crenezumab, solanezumab, donanemab, and lecanemab [6]. Aducanumab very recently received accelerated FDA approval on 7 June 2021 [7]. Donanemab has shown to improve the composite score for cognition and the ability to perform daily activities in clinical trials and has awarded FDA Breakthrough Therapy designation in June 2021 [8,9].

Previous studies have analyzed the relation between Aβ and Alzheimer’s to understand how the Aβ peptides aggregate and form plaques and how a drug can inhibit aggregation [3,10,11,12]. Other studies evaluate the different mutations of Aβ through different methods to generate a ranking of the different mutations based on their rate of aggregation. According to Tabaton et al., Aβ also plays a large signaling role for the BACE1 receptor. The signaling domain refers to Residues 1–8. There is a gap with the literature available in terms of the relation between aggregation and the signaling domain. This molecular simulation creates the opportunity to analyze these residues and evaluate the impact of the signaling domain on aggregation. No other molecular simulation provides the ability to specifically analyze the signaling domain. This means that this research is a novel approach to determine the importance of the signaling domain on aggregation.

This research evaluates both familial Alzheimer’s disease (FAD) and cerebral amyloid-angiopathy (CAA). These two are the diseases analyzed along with wildtype (WT) for the purposes of this research. There were also several different mutations that were analyzed that correspond to the diseases. These are A21G (Flemish), A42T (C-terminal), D7N (Tottori), D23N (Iowa), E22G (Arctic), E22K(Italian), E22Q (Dutch), L34V (Piedmont mutation), and WT (wildtype). These mutations were analyzed in the methods below. There were three main methods used to derive the conclusions. These methods all analyzed the residues of Aβ and tried to identify the important regions determined.

This research applies machine learning to the ensembles of conformations of Aβ variants to identify which specific amino acid residues undergo structural changes that can be related to disease severity and Aβ aggregation. These structural changes were accounted for in the data set shown by variance in the angles. By analyzing the changes and the corresponding result of the mutations and thus diseases, an overall conclusion can be derived about the region of Aβ that needs to be targeted for more research. To complete the research, the machine learning algorithm helped rank angles and determine the important residues.

## 2. Materials and Methods

### 2.1. Molecular Simulations

Molecular dynamics (MD) simulations were used to explore different conformations of the Aβ42 mutants and wild-type peptides. Because Aβ is intrinsically disordered, the conformational ensemble could not be quantified using global structural changes as with calmodulin [13]. Instead, the phi-psi angles of the peptide backbone were used as the features to characterize variant-specific conformational ensemble. Variants were modified using the VMD mutation tool, through deletion and insertion of variant residues into the wild-type Aβ42.

To generate random independent initial structures for wild-type and mutant peptides, we performed preliminary nanosecond simulations at 600 K starting with the PDB structure 1IYT for wild-type Aβ42. These preliminary simulations were performed using NPT ensemble in explicit solvent and had the initial dimensions of the unit cell of 94 Ǻ × 93 Ǻ × 97 Ǻ. Non-bonded interactions were computed using a smooth switching function applied between 10 and 12 Ǻ. Particle-mesh Ewald summation with the grid size of 1 Ǻ was used for electrostatic interactions. Langevin dynamics with the damping coefficient of 10 ps^−1^ were employed for temperature control, whereas the pressure control was applied by using the Langevin piston method and coupled unit cell dimensions. The time step was set to 2 fs. The final structures in the preliminary simulations were used to initiate production 20 ns NVT MD simulations at 295 K. During these simulations non-bonded interactions were smoothly switched off in the interval from 7 to 8 Ǻ. Although we utilized the cut-offs for the switching function different from the CHARMM default values, we applied them uniformly for all Aβ variants. Consequently, we believe that any resulting differences are likely to cancel out when we compare the conformational ensembles of the WT and mutants. To improve sampling, three separate MD simulations were performed for each variant and wild-type Aβ. Please see https://github.com/MDPPM/initialCode (accessed on 1 April 2021) for all configuration file parameters.

It is important to determine if short 60 ns MD simulations can be used for differentiating Aβ mutants. Four arguments suggest affirmative answer. First, previous studies have noted that Aβ1-42 monomer requires 60 ns/replica equilibration in replica exchange explicit water MD simulations [14]. Therefore, our 60 ns cumulative constant temperature simulations are not expected to reach complete conformational convergence. However, we have evidence that our sampled Aβ1-42 structures start to approach a “true” conformational ensemble. To this end, we evaluated the consistency between the J-couplings computed from our wild-type Aβ1-42 simulations and the two sets of experimental data [15,16]. The respective correlation coefficients are 0.30 and 0.46, which are close to those reported by Rosenman et al. (ranging from 0.43 to 0.48) [17]. We also computed the root mean squared deviation (RMSD) between the two sets of experimental J couplings and our in silico respective values. The resulting RMSDs are 0.94 and 1.17 Hz, whereas Rosenman et.al reported them to be from 0.85 to 1.25 Hz. Thus, our Aβ conformational ensemble approaches the experimental structures and those sampled in previous replica exchange simulations. Consequently, when we compare the conformational ensembles of wt peptide against its mutants, we are likely to discern their genuine conformational differences. Second, we would like to emphasize that our analysis is relative rather than absolute, i.e., we compare wild-type and mutant peptides rather than exhaustively analyze each individually. In other words, our premise is that mutations introduce sufficiently strong biases in the free energy surfaces differentiating the structures of Aβ wild type and mutant species even within limited conformational sampling. Third, we computed the time dependence of structural RMSD, which evaluates the drift of MD sampling from the reference Aβ structure. We show in Supplemental Materials in Figure S1 that RMSD plot reaches baseline for wild-type Aβ peptide. Such RMSD behavior is a necessary condition for simulation convergence. Fourth, additional point supporting the robustness of our conclusions is that we partitioned the 60 ns sampling into several subsets and recomputed the lists of amino acids determining aggregation propensities. We did not observe significant differences in amino acid ranking between the entire dataset and its subsets.

The phi and psi angles obtained from the MD simulations were analyzed using the Orange3 Data Mining Software (https://orangedatamining.com/, accessed on 1 April 2021). The “Rank” widget used to score the angles the based on internal scorers such as information gained, Chi-squared, and linear regression (https://orange3.readthedocs.io/projects/orange-visual-programming/en/latest/widgets/data/rank.html, accessed on 2 September 2021). This work used the “information gained” scorer to rank the angles which is defined as the expected amount of information from the angles (entropy reduction). This module works by scoring the variables based on their correlation with each target variable as described below. This is based on different scorers in relation to their class. In this situation, the module works by scoring the different angles in relation to the disease/mutation. The “test and score” module analyzes the angles with their ability to account for the data under the ROC curve. For this, 66 percent of the data is the training data leaving 33 percent for the testing. This module required a learning signal. This research used a random forest with five trees for this analysis. Finally, the “confusion matrix” was used to determine accuracy scores. This allows the research to test the conclusions generates to ensure that the conclusions are accurate.

### 2.2. Training Data Sets

The data set was generated from the MD molecular simulations as described above and in [13]. These data provide over 38,000 combined structural snapshots for the torsion angles of wild-type and variant Aβ and the mutation and disease they correspond to. The data set can be characterized by either mutation or disease. Both choices have their own benefits.

Benefits of ranking by disease: There are only three variables that the data need to be characterized by. Furthermore, it is the final phenotypic expression of the data. This means that it is observable and can be used by the data to make observations and check for errors.

Benefits of ranking by mutation: This is a lot more specific as the data can target many more variables that can be used to determine important residues. Furthermore, it is much easier to determine mutations that aggregate faster meaning the model can account for increased or decreased aggregation mutations to check important residues.

To accurately train the data, the research made each angle a “feature” that can be used to develop the model. Depending on what the data were being trained to test, the mutations and disease were either a “target” or “meta” for this analysis. This ensures that the data are purely characterized by the angles and will not be characterized by the mutations or disease.

## 3. Results

We analyze the contributions of different amino acid residues to measures of functional changes and disease for the different variants. These were performed using several machine learning methods to find the phi and psi angles that correlated with the different measures. These measures included Aβ aggregation, prediction of pathology, drugs targeting Aβ, and average age of onset. The results are shown with a table showing the rank and information gained for each angle. With this, plots were generated depicting the rank of the angles corresponding the residues they target. This allows for the visual understanding of the general location of the residues. Finally, a confusion matrix was generated to test for sensitivity and accuracy. This allows for a final understanding of the angles’ ability to characterize the disease. This allows us to determine whether the final angles generated can effectively characterize amyloid-beta mutations and Alzheimer’s disease. This helps check the work to check if the results make sense.

### 3.1. Prediction Amyloid β Aggregation

The first set of data mining analyses ranked the phi and psi angles of the ensemble of structures based on their ability to characterize measures of Aβ aggregation in the different variants. This research used the data set developed in our previous work as explained in the Methods Section titled “Molecular Simulations” for all the data mining results generated in the prediction of Aβ aggregation [13]. The variants studied both in vitro and in vivo experimentally by Hatami et al. were separated into two classes, variants that displayed increased aggregation (E22G, L34V, D7N, E22K, A2V, and H6R) and variants that displayed decreased aggregation (A21G and E22Q) [18]. The three variants that aggregated the most (E22G, L34V, and D7N) were used for the rankings. The “rank” learning module was used for developing a ranking of phi and psi angles involved in aggregation.

First, the ranking of angles in the variants that cause increased aggregation was compiled. These rankings are shown in Table 1 and in Figure S1 as a plot with the rank and residue as the axis. These results show how the eight of the residues in the top fifteen were part of the signaling domain (Residues 1–8) which is important for interactions with other molecules. Table 1 has the ranking of the angles with their information generated.

Similarly, Yang et al., observed in vitro that variants E22G, D23N, and E22Q aggregated faster than variants E22K, A21G, and WT [19]. Both papers are similar in that they both find mutations that aggregate faster, but the mutations they study differ. The research from Yang et al. evaluated the mutations that aggregated much faster than the ones that did not. From this paper, it was determined that similar to the last method, this method only ranks the mutations for both increased and decreased aggregation. This analysis also uses the data from the molecular simulations coupled with the “rank” module. Figure S2 and Table 1 show the results of the method developed based on this paper. Again, many of the angles ranking high were part of the signaling domain. Based on this method, five of the top fifteen ranking angles were in the signaling domain. Table 1 shows the ranking of the angles along with the information gained from each angle.

### 3.2. Prediction of Pathology

To determine significant residues associated with pathology an alternative analysis generates a classification tree using the “tree” and “test and score” module. The classification tree is a machine learning algorithm that works by dividing the data into subsets. It then predicts the results of the angles in figuring out the target variable with a percent score. The molecular simulation data used were the same data used above. The angles that could separate the WT from the non-WT points by >0 percent were compiled to form a Table 2 of angles that are able to predict variants of Alzheimer’s (FAD, familial Alzheimer’s disease and CAA, cerebral amyloid angiopathy) from wild type. These are only the angles that can separate WT from the FAD and CAA variants, but not FAD from CAA. These angles indicate a structural change in the Aβ peptide which might result in some functional change that is associated with disease.

Table 3 was then used to rank the entire data set by disease to determine the most useful angles that are the best at characterizing disease. Figure S3 is the result of the ranking of the entire data set by variant of disease based on the angles that could separate disease first. These are the angles that were best able to characterize the data as FAD, CAA, or WT. For the ranking of disease, the fifteen angles that ranked the best were angles phi6, phi8, Table 3 has the angles and their information gained generated from this method.

One more method was also applied for this research to predict Aβ pathology. This set of data mining analyses ranked the ensemble of phi and psi angles based on their ability to characterize the entire data set based on mutation and disease. These angles may be significant since they are the most capable in classifying the mutation or disease. This research characterizes both mutations and diseases to ensure that the angles that are the most useful in predicting the important residues are considered. Figure S4 and Table 4 are the result of the ranking of the entire data set by variant of disease. These are the angles that were best able to characterize the data as FAD, CAA, or WT. Figure S4 has five angles that are ranked as part of the top fifteen in the signaling domain. These angles include phi2, phi4, phi7, phi5, and psi3. Table 4 has the ranking of the angles and information gained for this method. Table 5 and Figure S5 are the result of the ranking of the entire data set by variant of mutation. These are the angles that were best able to characterize the data as the specific mutation that causes the disease. This ranking result shows five angles that were part of the signaling domain that ranked extremely well. These angles in the signaling domain are phi2, phi4, phi7, phi5, and psi3. Table 5 and Figure S5 show the angles that rank the entire data set by mutation and the information gained. In this ranking, eight of the fifteen angles that ranked best are part of the signaling domain. These angles are phi2, phi6, phi8, psi5, phi7, psi2, phi4, and psi1.

### 3.3. Average Age of Onset

The final method looks at the average age of onset for the mutations of Aβ mutations. With this, there are some mutations that have a significantly faster average age of onset compared to others. This may be related to the aggregation of Alzheimer’s disease as mutations with a lower age aggregate faster. Using this, the mutations that have an average age of onset of less than 60 were analyzed and the 84 angles were ranked based on their ability to influence these mutations. This is important as these angles might be important in the aggregation of Aβ. These angles were then analyzed to determine the important residues in characterizing mutations with a large average age of onset.

This research tests to see the angles that are significant in characterizing mutations with an average age of onset that are less than 60 years old. The mutations and their average age of onset was found through the paper McCoy et al. [13]. This method applied the “rank” module again to test the angles. Table 6 refers to the average age of onset along with the mutation. In this, we can see four of the mutations causing FAD and four of them causing CAA. Four of the mutations have an average age of less than 60 years old.

The results are shown in Table 7 and Figure S6. These are the angles that are best able to characterize the mutations with an average age of onset less than 60 years old. There are six angles that are ranked in the top 15 that are also part of the signaling domain. These angles are phi4, psi3, psi5, psi1, psi4, and phi5. Table 7 has the information gained and the rank of angles for this process.

### 3.4. Cumulative Ranking of Residues

Based on the results generated above, for each ranking, the fifteen angles that have the highest rank were given a point value equivalent to their fifteen minus their rank (16-ranking position). For methods where both increased aggregation and decreased aggregation were calculated, only increased aggregation was used for the purposes of this research. This generates seven unique tables with point values.

For the drugs in clinical trials, since residues were not ranked, a new part of the algorithm was utilized. This created a system where every time a residue was included in a clinical trial, it would gain five points. If a residue was targeted more than once, it would gain five times the number of times it was repeated points.

**Figure 1:**
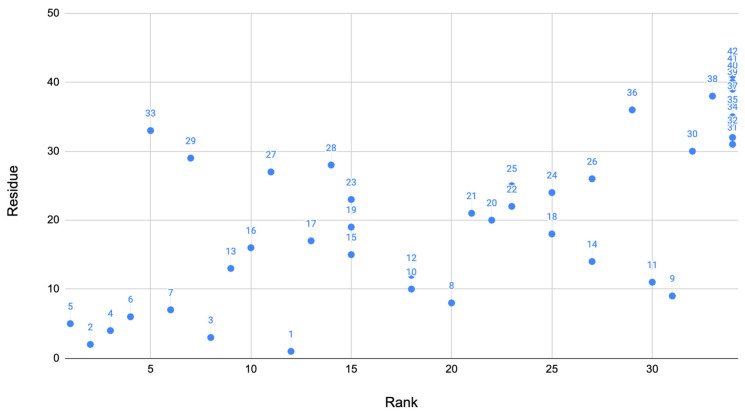
Overall ranking: Using the algorithm developed, a dot plot was created for the rank compared to the residue. This dot plot contains the final results of the methods combined. As shown, the signaling domain contains many of the key residues. Each point is labeled with the residue number.

These two methods of the algorithm produce scores for residues that were important for each method. From this, the scores are all added up for the final system where we can determine the most important residues. These scores of the residue were then plotted against the residue as a visual showing the residues scores compared to others. The rank of the residue was also plotted against the residue to show how the residues ranked in comparison to each other (Figure 1). Based on this algorithm described above, the 10 residues that ranked best are: 2, 3, 4, 5, 6, 7, 13, 16, 29, and 33. Table 8 shows the ranking of the residues above. This suggests that the residues in the range 2–7, 13, 16, 29, and 33 were the most important in the Alzheimer’s disease phenotype. This graphs clearly shows that many angles found in the signaling domain are highly ranked (bottom left corner of the plot). A few other residues also rank well in other key locations of the protein.

**Figure 2:**
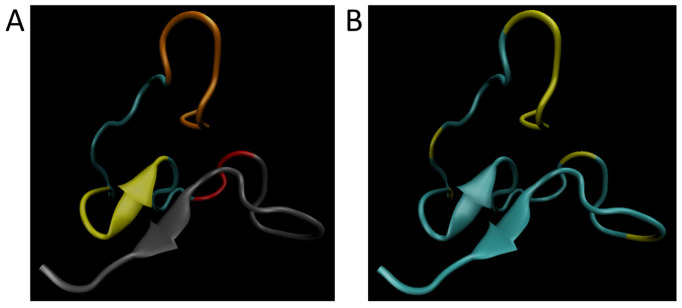
Image of residues on Aβ. (**A**) Simulated wild-type structure of Aβ showing the signaling domain in the N-terminus (Residues 1–8, orange), the hydrophobic cluster (Residues 17–21; yellow), the hairpin turn region (Residues 27–29; red), and the oligomerization domain in the C-terminus (Residues 30–42; gray). (**B**) Simulated wild-type structure showing the significant residues (Residues are 2–7, 13, 16, 29, and 33) predicted by this research have been highlighted in yellow.

Figure 2A shows the simulated wild-type structure of Aβ with the signaling domain in the N-terminus (Residues 1–8, orange), the hydrophobic cluster (Residues 17–21; yellow), the hairpin turn region (Residues 27–29; red), and the oligomerization domain in the C-terminus (Residues 30–42; gray). Figure 2B has the simulated wild-type structure showing the significant residues (residues are 2–7, 13, 16, 29, and 33) predicted by this research have been highlighted in yellow. From this it becomes clear that the signaling domain and hairpin turn are well represented in the residues for ranking by cumulative disease phenotype.

**Figure 3:**
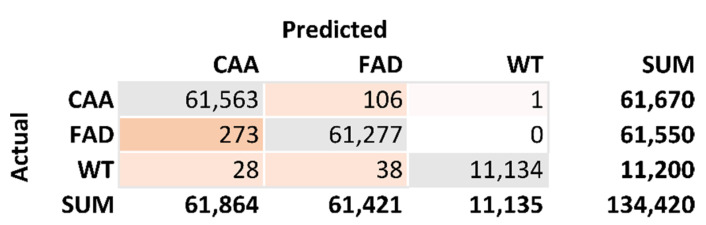
Confusion matrix of results. This generates this classification tree which is characterized for disease. The model trained on 66.67% of the data and tested the last 33.3%. This is used to produce an accuracy of 99.67%. This was also used to calculate specificity and sensitivity of the results.

From these models, a confusion matrix was developed to check the accuracy of the model. Figure 3 is the confusion matrix generated from the 10 top-ranking residues. The total number of correct predictions is 134,064. The total number of incorrect predictions is 446. This results in an accuracy calculation of 99.67%. The sensitivity calculation for FAD is 0.9983. The sensitivity calculation for CAA is 0.9966. The sensitivity calculation for WT is 0.9941. The specificity calculation for CAA is 0.9958. The specificity calculation for FAD is 0.9980. The specificity calculation for WT is 1.0000.

## 4. Discussion

### 4.1. Novelty of Method

Machine learning is a relatively new idea for predicting torsion angles. Machine learning has been used to predict torsion angles since 2012 [20]. The torsion angles have also been able to predict the folding of different proteins before [21]. For Aβ specifically, torsion angles have been used to predict gene severity [13]. The methods used here are still very unique as currently no methods produced have been able to use torsion angles to predict the significant residues of different proteins. This is a unique method for Aβ prediction to discover new targets for different drugs.

### 4.2. Residues Involved in Aggregation

Evaluating the changes in the phi/psi angles is important in understanding locations where functional change may occur. The use of machine learning to find these functional loci presents a novel approach to identify important sites with applications such as the identification to which effective drugs can be targeted. This research evaluates the phi/psi angles that are best correlated with aggregation as well as those correlated with the signaling domain.

Based on our research, regions of Aβ such as the hairpin turn region (Residues 27–29) and the signaling domain (Residues 1–8) seem rank highly in separating the aggregation data of Hatami and Yang [18,19]. In general, the central hydrophobic cluster (17–21) and C-terminus (29–42) are believed important for aggregation [22,23,24]. For example, Residues 41 and 42 drastically speed up aggregation compared to Aβ1-40 [11]. Our analysis suggests that Residues 16–21 and 29–42 are less highly ranked as other possible loci where interactions between Aβ might occur during oligomerization. It might be that the highly ranked residues might help position the residues involved in oligomerization. Furthermore, other regions such N terminus (Residues 1–15) which are thought to interact with small, charged molecules have other functional implications [25,26]. It seems likely that cell signaling helps set up the cellular conditions to promote Aβ aggregation. This is important to analyze to figure out where all the main interactions are and what they can lead to. NMR structures also show that the changes in the C-terminus increases the chances of aggregation.

It is important to comment on the plausible mechanistic relationship between monomeric conformational ensembles and aggregation propensities. It has been shown that monomeric structures of Aβ species displaying different aggregation propensities are distinct. For example, Sgourakis et al. have studied Aβ1–40 and Aβ1–42 conformational ensembles by NMR and microsecond REMD [15]. Aβ1–42 aggregates orders of magnitude faster than Aβ1–40 and, consequently, they found clear differences in the C-terminus conformations manifested by more rigid structure in Aβ1–42 species. Thus, the “signals” differentiating aggregation propensities might be discerned even in monomeric conformational propensities. In our study, we have demonstrated that certain sequence positions strongly contribute for discriminating aggregation rates of Aβ mutants by serving as possible nucleation centers. It is then natural to expect that when these “important” amino acids are altered by mutations aggregation pathways are affected. However, mutations also affect monomeric conformational preferences, which are detected by our analysis and linked to aggregation rates. These arguments make up the premise of our approach.

### 4.3. Implications for Drug Targeting

There are many implications for future drug targeting. As the phi and psi angles involved in drug action that aims to reduce the aggregation of Aβ were analyzed. These drugs are all currently in clinical trials to test if they are effective. This research only looked at the residues for drugs that were in clinical trials since at least 2018. This is to ensure that the research only looks at recent drugs. This is important because this will result in the highest level of accuracy. This is because they use the most up to date research. Furthermore, they have not been proven to fail yet as the other drugs have.

Analysis of these drugs is critical as it allows us to understand research that is not available. The drugs would only target the residues if past researchers found that there may be some significance at those regions. This must mean that previous research found that these residues were significant. This is because the drugs would not target these locations unless they felt that changing something at these residues would decrease the aggregation. This means that these residues may provide some functional change at the locations. This is important to ensure that the residues are important in figuring out the significant locations.

These are the drugs that aimed at reducing the aggregation of Aβ that made it to clinical trials. For the purposes of this research, we looked at the drugs that are still in clinical trials. This refers to aducanumab, gantenerumab, crenezumab, solanezumab, donanemab, and lecanemab. These drugs will have their residues analyzed for these models. The residues targeted were found from past literature available for Aβ.

This method essentially analyzes all the residues that are targeted by drugs in clinical trials. These residues were then scored based on frequency. Table 9 lists the drugs in clinical trials and the residues they target. This was used to identify important locations and domains. The drugs in clinical trials target Residues 3–11 and 13–27. Drugs that failed clinical trials were also analyzed based on their function and their result. In some cases, the exact residues targeted by these drugs are not publicly available. The drugs that failed, mechanism class, and results have been compiled into Table 10. The anti-tau antibodies and secretase inhibitor drugs shown in Table 1 have not successfully completed clinical trials. This table can be used to further evaluate the residues if the knowledge becomes public [27].

Based on the cumulative ranking of important resides presented here, drugs that target Residues 27–29 and/or 2–8 will be effective in reducing symptoms of Alzheimer’s. Based on this, aducanumab, donanemab, and gantenerumab seem to be the drugs that will be most effective in reducing aggregation. This prediction is supported by the FDA approval of aducanumab and favorable findings in current trials for gantenerumab [7,28]. Recent reports indicated that Alzheimer’s patients taking donanemab showed improvement over those taking placebo [8,9]. This analysis suggests that the reason that the drugs targeting Aβ in clinical trials have failed and will fail in the future is because the drugs are not targeting the regions of Aβ that have the largest effect on disease phenotype.

## 5. Conclusions

Applying machine learning to ensembles of conformations from REMD simulations proves a new perspective on how to evaluate which residues are involved in disease phenotypes arising from variants to Aβ. The cumulative ranking suggests that Residues 27–29 in the hairpin turn and Residues 2–8 in the signaling domain at the N-terminus are the most important for this separation. This means that they have structural changes as indicated by the phi and psi angles that can differentiate between the different phenotypes of different variants. The correlation of these rankings with phenotype create the potential to identify the residues involved, with disease severity, aggregation and drug action.

## Figures and Tables

**Table 1:** Backbone Angles Ranked Based on Aggregation.

Hatami et al. Data.		Yang et al. Data	
Angles	Information Gain	Angles	Information Gain
psi27	0.793	psi5	0.585
phi6	0.793	psi1	0.567
phi5	0.725	phi29	0.47
psi5	0.688	psi27	0.446
phi2	0.673	phi4	0.434
psi1	0.656	psi27	0.422
psi28	0.641	phi17	0.387
psi15	0.598	psi28	0.384
psi6	0.59	phi5	0.358
phi19	0.577	phi25	0.356
phi3	0.556	phi2	0.349
psi13	0.529	psi16	0.304
phi10	0.529	psi19	0.28
psi7	0.504	phi30	0.274
phi22	0.497	phi36	0.27

These are the 15 angles that were predicted to be the most significant based on Hatami et al. (left) and Yang et al. [19] (right). The three mutations that aggregate fastest were used and the angles [18] that best rank these are shown.

**Table 2:** List of Dihedral Angles Separating Disease Phenotype Based on Classification Tree.

psi2	psi6	phi11	psi16	phi31
psi3	phi7	psi12	psi23	phi33
phi3	psi7	psi13	phi25	phi36
phi4	psi9	phi15	phi28	psi32
phi5	phi8	psi15	psi27	psi39
phi6	phi10	phi16	psi30	

The angles capable of separating WT from FAD and CAA generated from the classification tree with the Orange3 data mining software. The angles in this table are listed starting at the N-terminus.

**Table 3:** Results of Ranking Data Set Capable of Sorting by Variant by Disease.

Angles	Information Gained
phi6	0.821
phi8	0.724
phi7	0.708
phi33	0.647
phi16	0.63
psi2	0.621
psi13	0.613
psi28	0.571
phi4	0.657
psi27	0.56
psi13	0.559
psi16	0.556
phi15	0.553
phi10	0.535
psi9	0.51

These 15 angles that are predicted to be most significant were used for the analysis. This is based on a ranking of how well they characterize disease.

**Table 4:** Results of Ranking Entire Data Set by Disease.

Angles	Information Gained
phi2	0.232
phi33	0.215
phi29	0.212
phi4	0.19
phi7	0.183
psi29	0.181
psi12	0.178
psi13	0.159
phi5	0.159
psi3	0.154
psi14	0.148
psi10	0.147
psi21	0.146
psi38	0.144

These 15 angles that were predicted to be the most significant based on how well they ranked the entire data set by disease were used for analysis.

**Table 5:** Results of Ranking Entire Data Set by Mutation.

Angles	Information Gained
phi2	0.85
phi6	0.821
phi8	0.724
psi5	0.711
phi7	0.708
phi33	0.647
phi16	0.63
psi2	0.621
psi13	0.613
phi29	0.597
phi17	0.578
psi28	0.571
phi4	0.567
psi27	0.56
psi1	0.559

These are the top 15 angles that were predicted to be the most significant based on how well they ranked the entire data set by mutations. These top angles were ranked and the top 15 were used for analysis. A total of eight of the fifteen angles are part of the signaling domain.

**Table 6:** Average Age of Onset.

Disease	Mutation	Average Age of Onset
Familial Alzheimer’s Disease	A21G	46.2
Familial Alzheimer’s Disease	A42T	63.0
Familial Alzheimer’s Disease	D7N	60.0
Familial Alzheimer’s Disease	E22G	57.5
Cerebral Amyloid Angiopathy	D23N	68.9
Cerebral Amyloid Angiopathy	E22K	55.0
Cerebral Amyloid Angiopathy	E22Q	55.0
Cerebral Amyloid Angiopathy	L34V	60.0
Wildtype	WT	65.0

These are the average age of onset for each of the mutations of Aβ used in the data set. The mutations that have an average age of onset less than 60 years were used for this analysis to predict the residues that aggregate faster.

**Table 7:** Results of Ranking by Results Based on Average Age of Onset.

Angles	Information Gained
phi29	0.519
phi4	0.439
psi3	0.382
psi5	0.381
phi10	0.359
phi17	0.336
psi23	0.297
phi33	0.241
psi33	0.236
psi1	0.229
psi4	0.223
psi30	0.208
psi15	0.208
psi20	0.205
phi5	0.204

These are the 15 angles that were predicted to be the most significant based on how well they ranked the mutations that have an average age of onset less than 60 years. The top angles were ranked and the top 15 were used for analysis.

**Table 8:** Conclusion of Best Ranking Angles.

Rank	Residue
1	5
2	2
3	4
4	6
5	33
6	7
7	29
8	3
9	13
10	16

The cumulative ranking of the 10 residues that were predicted to be significant.

**Table 9:** Drugs in Clinical Trials and Residues Targeted.

Name of Drug	Amyloid β Residues Targeted
aducanumab	3–7 (6, 25)
gantenerumab	3–11 and 18–27 (6, 25)
crenezumab	13–24 (6, 25)
solanezumab	16–26 (6, 25)
lecanemab	1–16 (6, 25)
donanemab	3–7 (25)

These drugs are in a stage of clinical trials and the residues they target are also shown with them. This was used for the analysis of the residues as it indicates what other researchers predicted significant residues are.

**Table 10:** Failed Anti-amyloid Drugs.

Drug	Mechanism of Action	Results
Semagacestat	Anti-amyloid–γ-secretase inhibitor (23)	Lack of Efficacy
Avagacestat	Anti-amyloid–γ-secretase inhibitor (23)	Lack of Efficacy
Tarenflurbil	Anti-amyloid-γ-secretase modulation to make less toxic form of amyloid β (23)	Lack of Efficacy
Lanabecestat	Anti-amyloid–beta-secretase 1 cleaving enzyme (BACE) inhibitor (23)	Lack of Efficacy
Verubecestat	Anti-amyloid–beta-secretase 1 cleaving enzyme (BACE) inhibitor (23)	Lack of Efficacy
Atabecestat	Anti-amyloid–beta-secretase 1 cleaving enzyme (BACE) inhibitor (23)	Lack of Efficacy
Bapineuzumab	Anti-amyloid–binds N-terminal region of amyloid β at Residues 1–5 (25, 26)	Lack of Efficacy
Solanezumab	Anti-amyloid–bind amyloid β at Residues 16–26 (6, 25)	Lack of Efficacy
Gammagard Liquid	Anti-amyloid antibodies (23)	Lack of Efficacy
LMTM	Anti-tau aggregation (23)	Lack of Efficacy
ponezumab	Anti-amyloid–bind amyloid β at Residues 30–40 (6, 25)	Lack of Efficacy

These are the past drugs that have failed clinical trials. This includes the mechanism of the drug and the reason for failure. This provides background into the drugs of amyloid beta.

## Data Availability

Data will be made available at the Mason Archival Research Service before publication (mars.gmu.edu).

## Supplementary Materials

The following are available online at https://www.mdpi.com/article/10.3390/biom11101541/s1, Figure S1: Results of Ranking by Results of Hatami et al., Figure S2: Results of Ranking by Results of Yang et al., Figure S3: Results of Ranking Data Set Capable of Sorting by Variant by Disease, Figure S4: Results of Ranking Entire Data Set by Disease, Figure S5: Results of Ranking Entire Data Set by Mutation, Figure S6: Results of Ranking by Results Based on Average Age of Onset.
