# Supplementary material for: Data Mining of Molecular Simulations Suggest Key Amino Acid Residues for Aggregation, Signaling and Drug Action
Source: Biomolecules. 2021 Oct 19;11(10):1541. doi: 10.3390/biom11101541 (PMC8534076; doi:10.3390/biom11101541)
Supplement: Supplementary file 1 [file biomolecules-11-01541-s001.zip › biomolecules-1359635-supplementary.pdf]

Supplemental Material

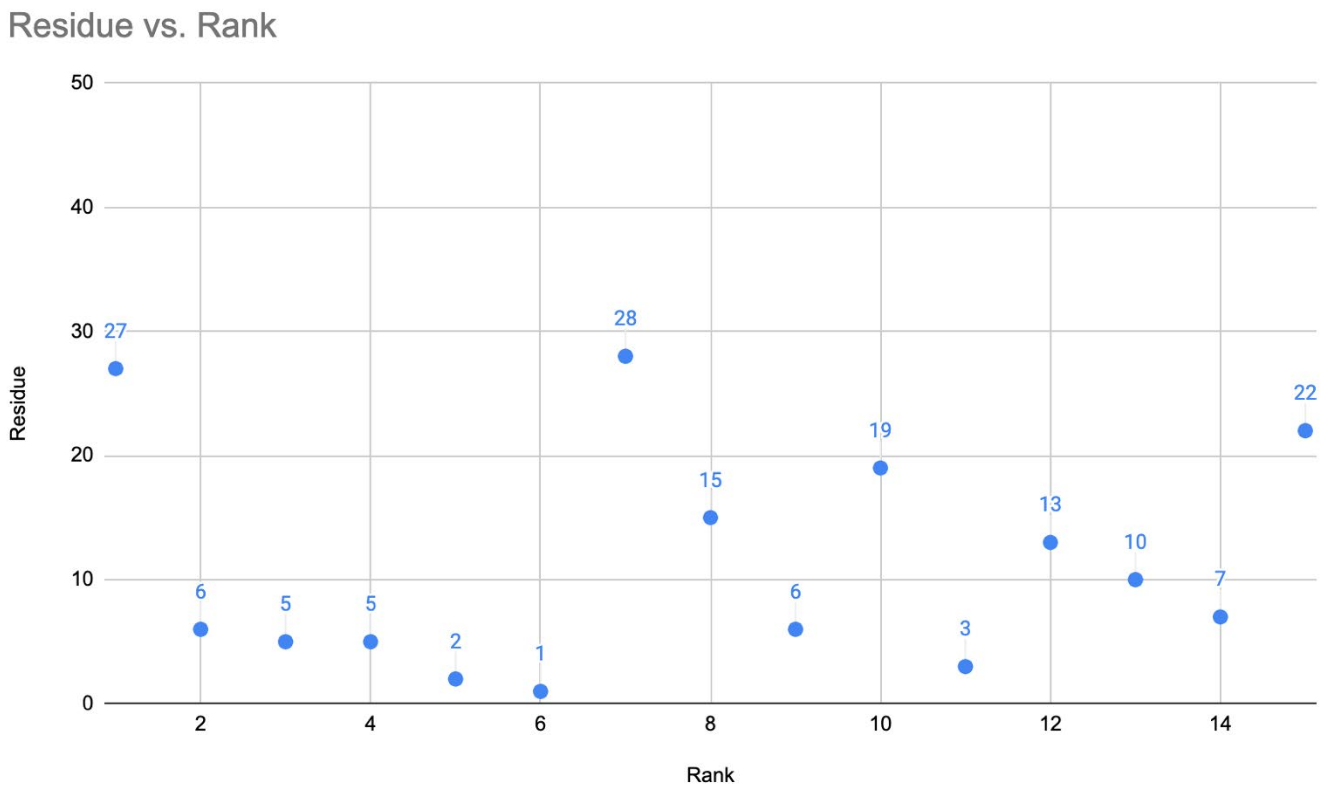

Figure S1 - Results of Ranking by Results of Hatami et al. The results of the ranking generated based on (12). Each point is labeled with the residue number. The mutations that aggregate faster (E22G, L34V, and D7N) were analyzed and the 15 residues that ranked these mutations best were plotted. 8 of these residues are part of the signaling domain.

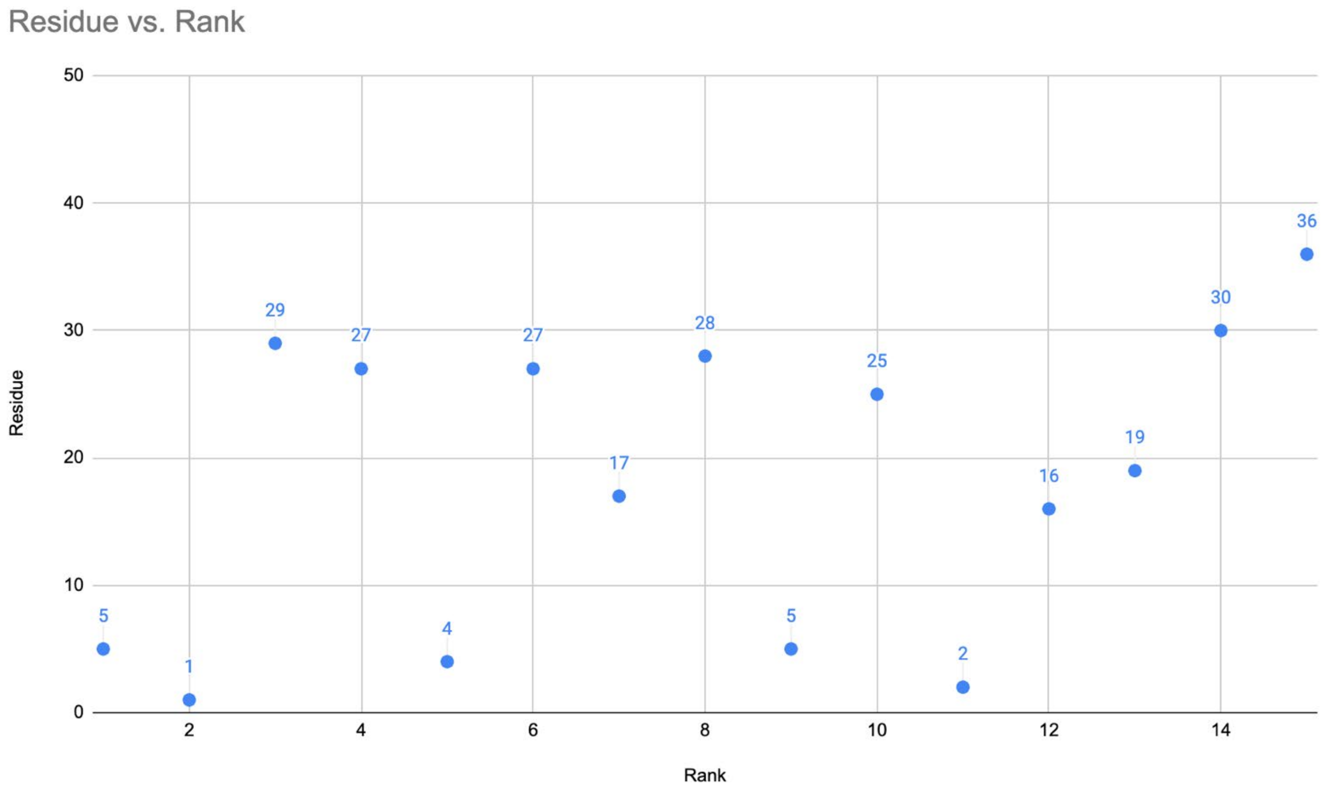

Figure S2 - Results of Ranking by Results of Yang et al. The results of the ranking generated based on (13). Each point is labeled with the residue number. The mutations that aggregate faster (E22G, D23N, and E22Q) were analyzed and the 15 residues that ranked these mutations best were plotted. 5 of these 15 residues are part of the signaling domain.

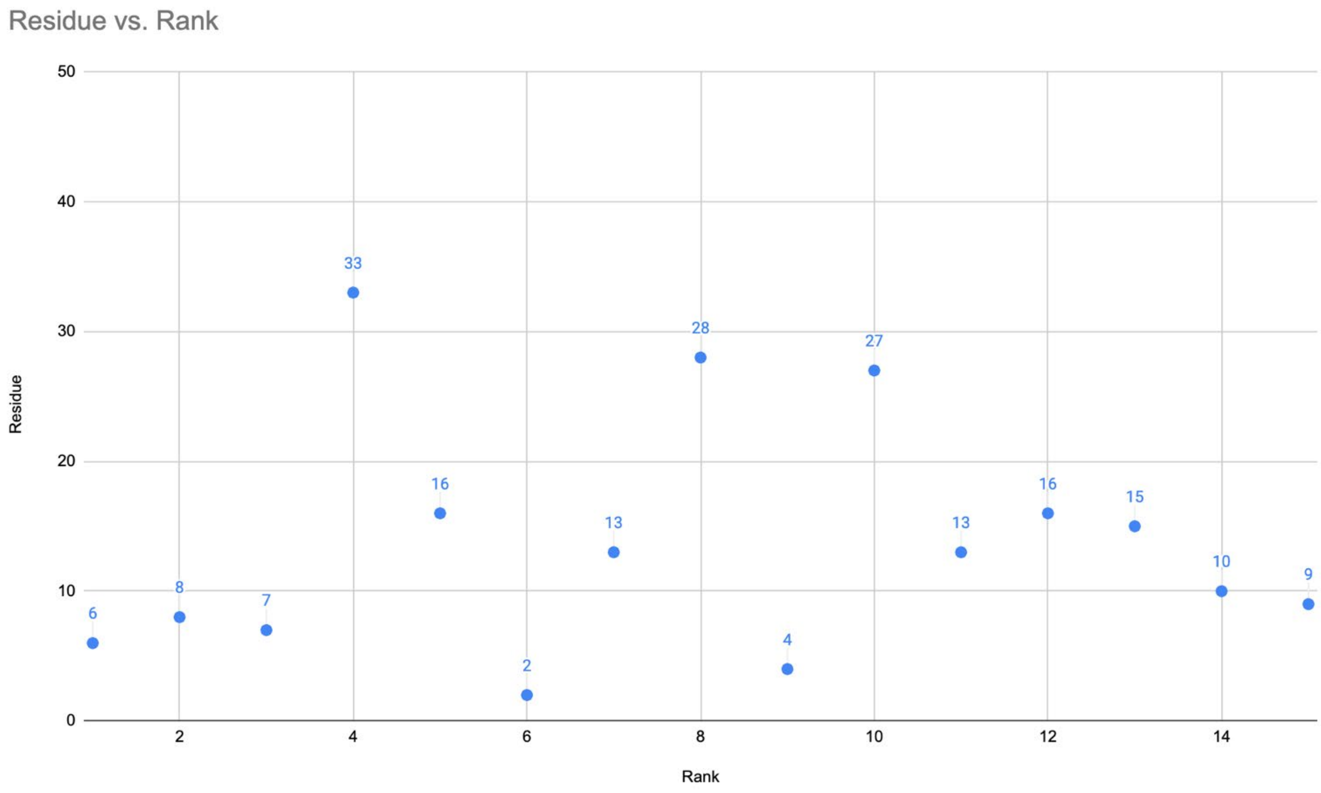

Figure S3 - Results of Ranking Data Set Capable of Sorting by Variant by Disease. Some of the residues are able to predict WT from non-WT variants. Each point is labeled with the residue number. These residues were compiled then ranked to determine the residues that are best able to rank the entire date set by the disease. This suggests that these residues may be significant as they can classify WT from FAD and/or CAA.

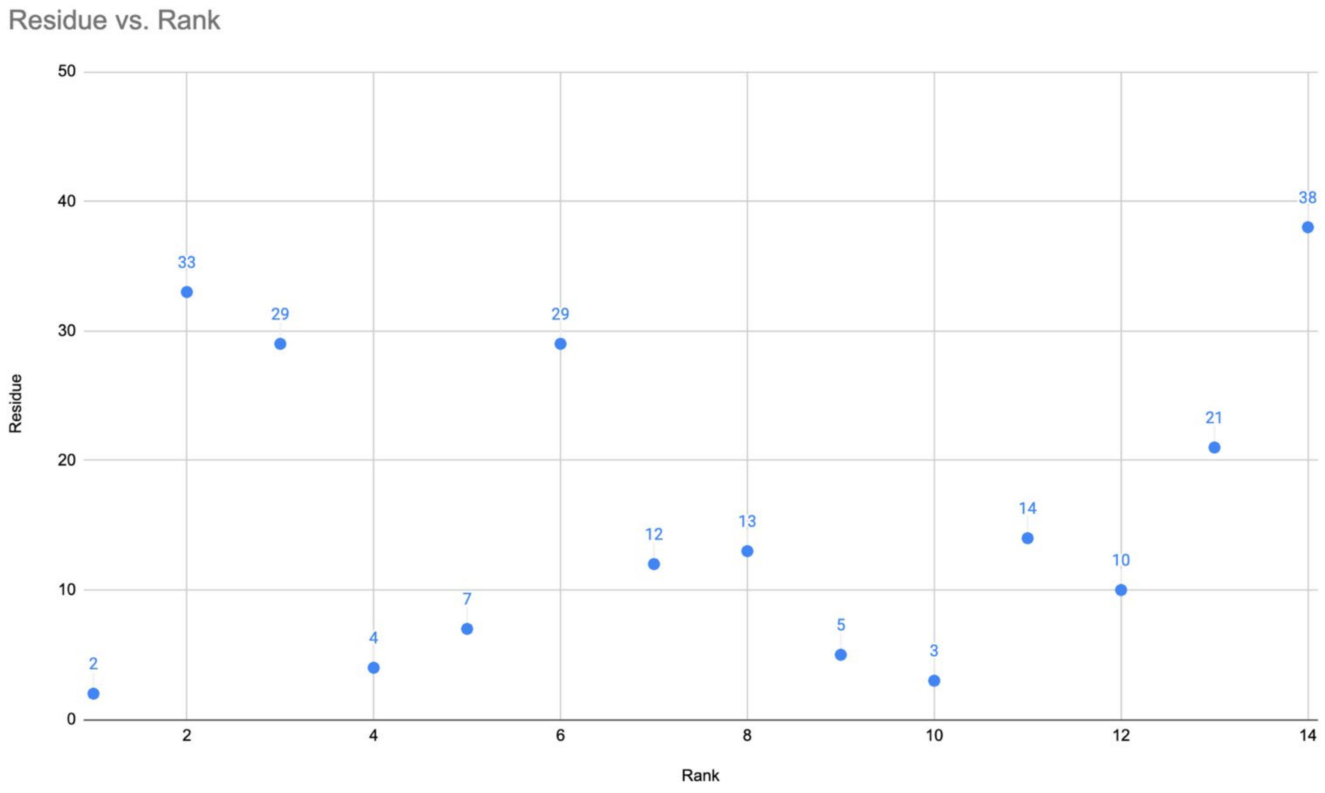

Figure S4 - Results of Ranking Entire Data Set by Disease. The entire set of angles was ranked based on their ability to rank the data set by disease. Each point is labeled with the residue number. Five of the residues are part of the signaling domain. This analysis predicts which residues that are the most important in predicting disease.

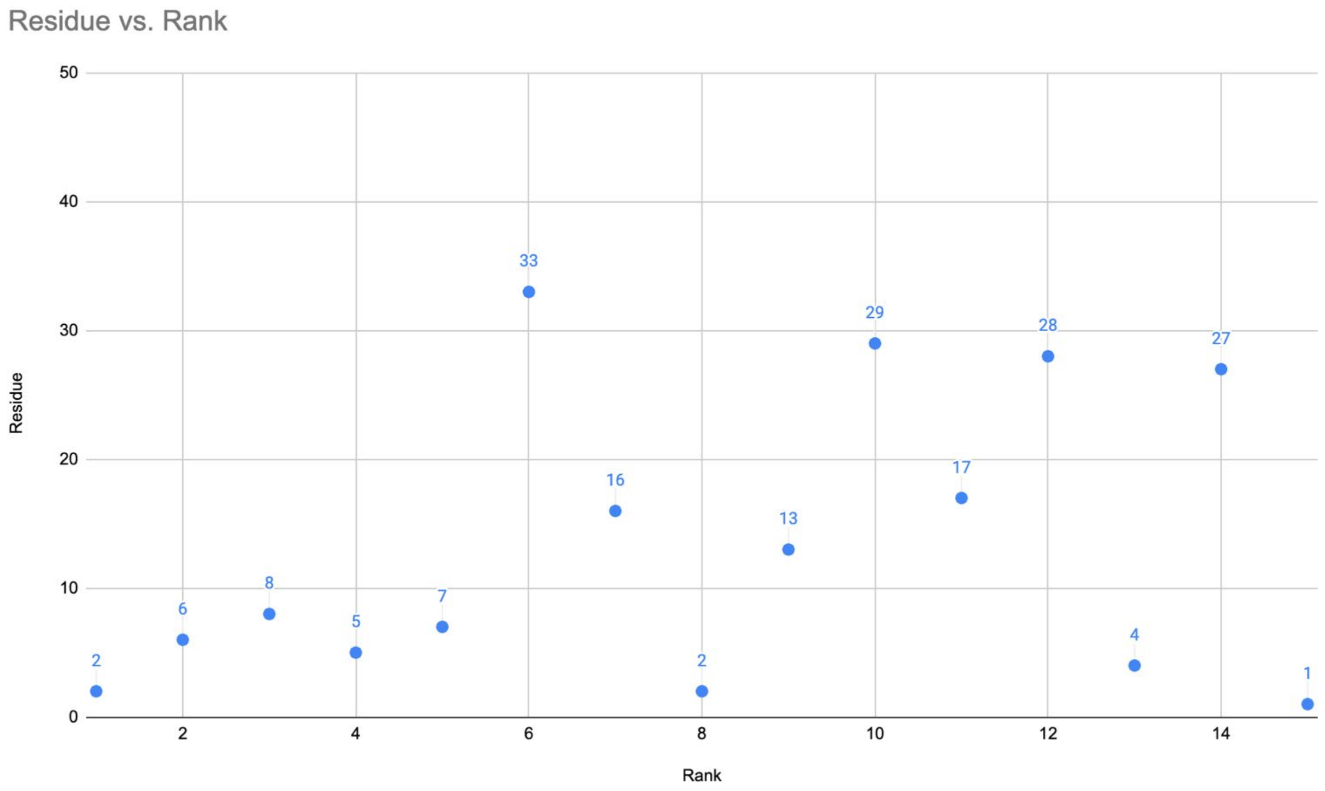

Figure S5 - Results of Ranking Entire Data Set by Mutation. The entire set of angles was ranked based on their ability to rank the data set by mutations. Each point is labeled with the residue number. Eight of the residues are part of the signaling domain. This analysis suggests the residues that are the most important in predicting mutations.

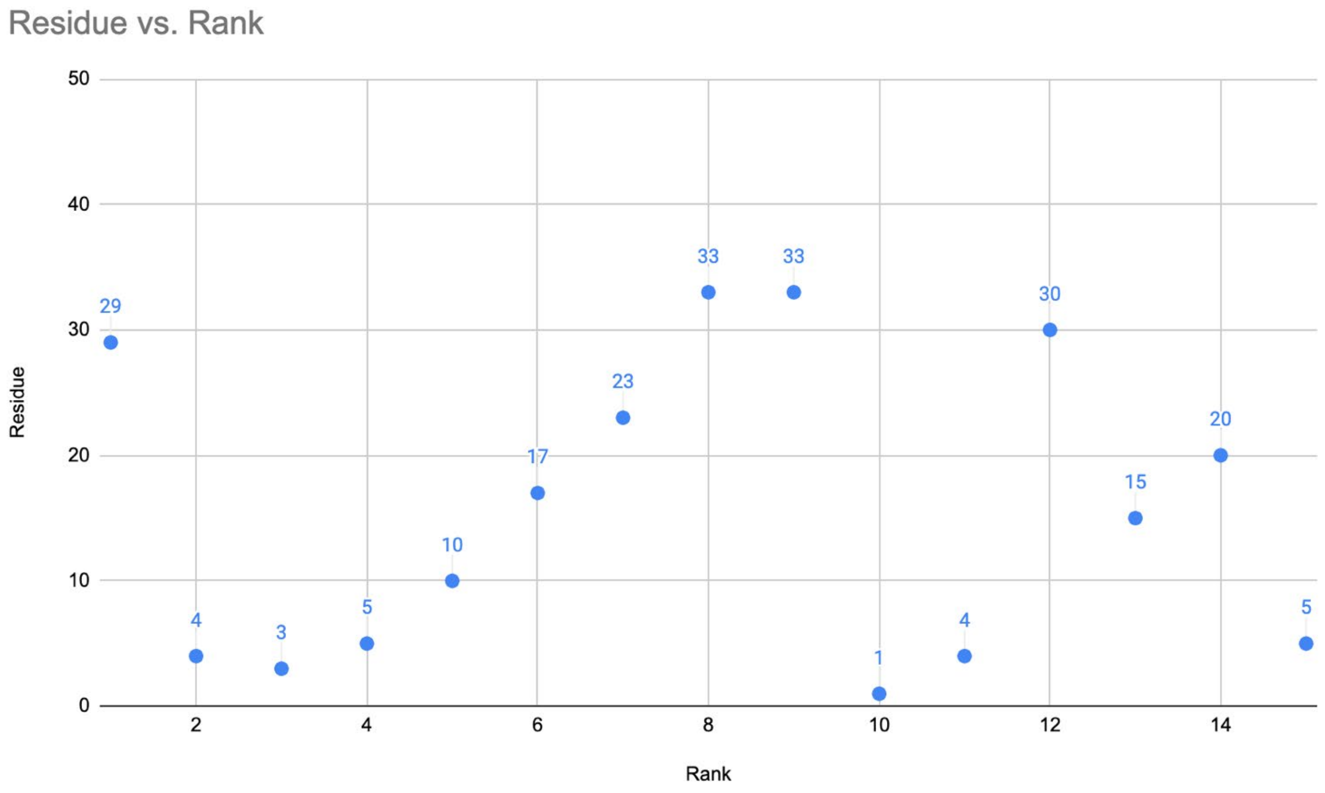

Figure S6. - Results of Ranking by Results Based on Average Age of Onset. The entire set of angles was ranked based on the mutations of amyloid-beta that have an average age of onset of less than 60 years. Each point is labeled with the residue number. The average age of onset is important because mutations with a younger average age of onset may aggregate faster. Six of the residues are part of the signaling domain.
